# *Cryptosporidium* spp. in captive snakes from 26 provinces in China: Prevalence, molecular characterization, and symptoms[Fn FN1]

**DOI:** 10.1051/parasite/2024047

**Published:** 2024-08-07

**Authors:** Yilei Zhang, Zhenxiao Lu, Lingru He, Guodong Xiao, Lijie Tian, Jiawei Zhu, Tian Liu, Qiangxin Ou, Haibo Chen, Yew Hwong, Yangjun Kang, Qianming Xu, Qingxun Zhang, Congshan Yang

**Affiliations:** 1 College of Animal Science and Technology, Anhui Agricultural University Hefei Anhui Province 230036 PR China; 2 Beijing Biodiversity Conservation Research Center Beijing 100076 PR China; 3 School of Forestry and Landscape Architecture, Anhui Agricultural University Hefei Anhui Province 230036 PR China; 4 Hengyuan Animal Hospital Hefei Anhui Province 230001 PR China; 5 Chongmu Pet Clinic Nanjing Jiangsu Province 211800 PR China; 6 Mengdele Pet Clinic Xiamen Fujian Province 361021 PR China

**Keywords:** *Cryptosporidium* spp., Snake, Molecular epidemiology, Symptom, China

## Abstract

Snakes are sometimes regarded as pets and are used in traditional Chinese medicine. *Cryptosporidium* spp. are frequently identified in snakes, representing an important pathogen and causing gastrointestinal diseases. Current data indicate that risk factors for infection and patterns of clinical symptom presentation may differ among *Cryptosporidium* spp. To better understand the infection status by *Cryptosporidium* spp., fecal samples were collected from 603 asymptomatic and 147 symptomatic snakes in 26 provinces of China. These samples came from *Elaphe guttata*, *Elaphe obsoleta, Pituophis melanoleucus*, *Thamnophis sirtalis, Lampropeltis getulus,* and *Heterodon nasicus.* The partial small subunit (SSU) rRNA gene was amplified using nested polymerase chain reaction (PCR) to investigate the infection rate of *Cryptosporidium* spp., and to assess evolutionary relationships and genetic characterization. A prevalence of 20% was recorded in asymptomatic snakes, with age identified as a significant risk factor. In contrast, 70% of symptomatic snakes were positive for *Cryptosporidium* spp., with *Cryptosporidium serpentis* and *Cryptosporidium varanii* (syn. *C. saurophilum*). Further analysis revealed a potential association between *C. serpentis* and regurgitation, and *C. varanii* and diarrhea, while neither species was linked to flatulence. To our knowledge, this is the first study to report *Cryptosporidium* spp. and associated clinical signs in symptomatic snakes in China. This study aims to enhance the understanding of *Cryptosporidium* infections, risk factors, and clinical manifestations in snakes, providing data crucial for the control and prevention of cryptosporidiosis.

## Abstract (fr)

Les serpents sont parfois considérés comme animaux de compagnie et sont utilisés en médecine traditionnelle chinoise. Des *Cryptosporidium* spp. sont fréquemment identifiés chez les serpents, ont un rôle d’agent pathogène important et provoquent des maladies gastro-intestinales. Les données actuelles indiquent que les facteurs de risque d’infection et les schémas de présentation des symptômes cliniques peuvent varier en fonction des espèces de *Cryptosporidium*. Pour mieux comprendre l’état d’infection par *Cryptosporidium* spp., des échantillons fécaux ont été collectés auprès de 603 serpents asymptomatiques et 147 serpents symptomatiques dans 26 provinces de Chine. Ces échantillons provenaient d’*Elaphe guttata*, *Elaphe obsoleta*, *Pituophis melanoleucus*, *Thamnophis sirtalis*, *Lampropeltis getulus* et *Heterodon nasicus*. Le gène de l’ARNr de la petite sous-unité partielle (SSU) a été amplifié à l’aide d’une réaction en chaîne par polymérase (PCR) imbriquée pour étudier le taux d’infection par *Cryptosporidium* spp. et évaluer les relations évolutives et la caractérisation génétique. Une prévalence de 20 % a été trouvée chez les serpents asymptomatiques, l’âge étant identifié comme un facteur de risque important. En revanche, 70 % des serpents symptomatiques étaient positifs à *Cryptosporidium* spp. avec *Cryptosporidium serpentis* et *Cryptosporidium varanii* (syn. *C. saurophilum*). Une analyse plus approfondie a révélé une association potentielle entre *C. serpentis* et la régurgitation, et *C. varanii* et la diarrhée, alors qu’aucune des deux espèces n’était liée aux flatulences. À notre connaissance, il s’agit ici de la première étude à signaler la présence de *Cryptosporidium* spp. et les signes cliniques associés chez des serpents symptomatiques en Chine. Cette étude vise à améliorer la compréhension des infections à *Cryptosporidium*, des facteurs de risque et des manifestations cliniques chez les serpents, en fournissant des données cruciales pour le contrôle et la prévention de la cryptosporidiose.

## Introduction

*Cryptosporidium* spp. is classified as a protozoan parasite of the apicomplexan group and is recognized as zoonotic [5, 41]. This genus has garnered significant attention globally due to its public health significance, and the World Health Organization (WHO) has identified it as an indicator of suspicion for AIDS [8, 30]. Cryptosporidiosis caused by *Cryptosporidium* spp. can be transmitted through the fecal-oral route, infecting the gastrointestinal tract of various vertebrates such as humans, domestic animals, birds, fish, and reptiles, either through direct contact or via ingestion of contaminated food or water [15, 16, 43, 49]. Cryptosporidiosis primarily manifests as acute gastrointestinal symptoms, which include prolonged watery diarrhea and abdominal pain [5, 29]. Additional symptoms may include fever, vomiting, and anorexia, among other clinical features. In immunocompromized patients, the infection can lead to severe, life-threatening conditions [5, 22, 48].

Snakes are popular pets globally and are valued for their role in traditional medicine and as a delicacy in Asian countries [2, 52]. Recent surveys indicate that over 1.7 million pet snakes are kept in American households [13, 42], while snake farming is rapidly expanding in Asian countries, with an annual trade volume of 9000 tons in China alone [2, 52]. Snakes as hosts of *Cryptosporidium* spp. and several neglected foodborne zoonotic diseases have been reported worldwide, such as in the Americas, Australia, Brazil, China, Italy, India, Japan, and Thailand [11, 28, 38, 39, 47, 48, 50].

To date, over 40 *Cryptosporidium* spp. and at least 70 genotypes have been identified in more than 90 countries [23, 40]. In snakes, *Cryptosporidium* frequently causes digestive disorders, often leading to chronic or occasionally lethal infections [13, 21]. Notably, *C. serpentis* and *C. varanii* are prevalent species in snakes, with numerous studies showing a disease-related distribution of these species [26, 34]. *Cryptosporidium serpentis* is dominant, leading to symptoms like anorexia, lethargy, postprandial regurgitation, mid-body swelling, and weight loss. Conversely, *C. varanii* primarily affects the intestines, causing enteritis and diarrhea, often without overt clinical signs. These infections are more common in adult snakes compared to juveniles, contrasting with the patterns observed in mammals and birds [7]. Additionally, although species such as *C. parvum*, *C. muris*, *C. baileyi*, *C. tyzzeri*, and *C. andersoni* are not associated with clinical symptoms in snakes, they pose a significant risk of human cryptosporidiosis upon contact with infected snakes [7, 29, 50].

Thus far, *Cryptosporidium*-infected snakes have implications for animal and public health and welfare [22, 37]. *Cryptosporidium* spp. infections in snakes, whether captive-bred or privately owned, have resulted in significant economic losses [6, 30, 31, 35, 42]. However, in China, little information is available about *Cryptosporidium* spp. infections in snakes compared to those in mammals and birds. Most molecular epidemiological studies on *Cryptosporidium* spp. infections in snakes have focused on asymptomatic samples, with few investigating the risk factors for cryptosporidiosis [11, 24, 28, 51]. Specific data on the effects of *Cryptosporidium* spp. on hosts and the related symptoms are limited. In this study, randomized surveys of asymptomatic snakes were initially conducted on farms, in pet stores, and in private households in China to determine the prevalence and genetic characterization of *Cryptosporidium* spp., as well as to assess associated risk factors. Symptomatic snakes were also considered in the investigation of *Cryptosporidium* spp. infections and related clinical signs. The potential associations between *C. serpentis* and *C. varanii* and their respective symptoms were assessed. This research aims to enhance our understanding of *Cryptosporidium* spp. infections, risk factors, and the clinical manifestations of the disease in snakes, providing preliminary data for the control and prevention of cryptosporidiosis.

## Materials and methods

### Ethical approval

This study adhered to the Guide for the Care and Use of Laboratory Animals set forth by the Ministry of Health, China. The protocol received approval from the Research Ethics Committee of the Anhui Agricultural University (number AHAUB2022017). Snake samples from private breeders, pet shops, and farms within the People’s Republic of China were included in this research. Prior to fecal sample collection, necessary permission was secured from the snake owners. Throughout the sample collection process, care was taken to ensure that the animals were not disturbed and stressed.

### Sample collection

**Figure 1:**
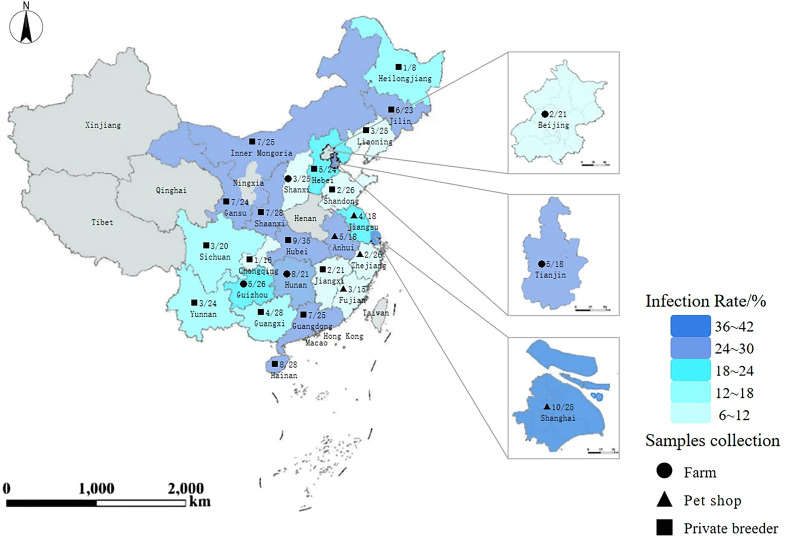
Map of China showing the specimen collection sites with circles, triangles, and squares, and the prevalence of *Cryptosporidium* spp. shown in different colors.

From July 2022 to December 2023, fresh fecal samples were collected randomly from six species and four age groups of snakes in China. These specimens were from various living conditions, including five farms, five pet shops, and sixteen private breeding sites (Fig. 1). On farms, about 600 free-ranging snakes were housed in a limited area, and 111 fecal samples were collected from separate sites. In pet shops and private breeding sites, all snakes of different ages were kept in individually covered plastic cages, preventing direct contact between them. From each cage, one fecal sample was collected, totalling 492 fecal samples. Altogether, 603 fecal samples were gathered across age groups: 0–1 year (*n* = 252), 1–2 years (*n* = 139), 2–3 years (*n* = 80), and ≥3 years (*n* = 132). At the time of collection, each was meticulously catalogued by age, sex, and species (Table 1). None of the animals showed any gastrointestinal clinical symptoms.

**Table 1:** Prevalence of *Cryptosporidium* spp. infection by sex, species, age, and living conditions. A potential risk factor for infection was considered when *p* < 0.05 and OR > 1.

Variable	No. Samples	*Cryptosporidium* spp.		
		No. Positive (%)	OR (95% CI)	*p*-value
Sex				
Male	274	61 (22.26)	1.294 (0.856–1.958)	0.222
Female	287	52 (18.12)	0.773 (0.511–1.169)	0.222
Unknown	42	9 (21.43)		
Species				
Corn snake (*Elaphe guttata = Pantherophis obsoletus*)	107	25 (23.36)	1.254 (0.761–2.067)	0.375
Pine snake (*Pituophis melanoleucus*)	95	15 (15.79)	0.703 (0.389–1.269)	0.242
Common garter snake (*Thamnophis sirtalis*)	87	17 (19.54)	0.951 (0.537–1.684)	0.862
King snake (*Lampropeltis getulus*)	105	23 (21.90)	1.130 (0.677–1.887)	0.639
Hognose snake (*Heterodon nasicus*)	118	22 (18.64)	0.882 (0.528–1.473)	0.632
Black rat snake (*Elaphe obsoleta = Pantherophis obsoletus*)	91	20 (21.98)	1.132 (0.659–1.946)	0.653
Age(years)				
0–1	252	44 (17.46)	0.740 (0.491–1.117)	0.152
1–2	139	19 (13.67)	0.555 (0.326–0.944)	0.030^*^
2–3	80	18 (22.5)	1.164 (0.660–2.052)	0.599
3+	132	41 (31.06)	2.169 (1.398–3.367)	0.001^**^
Living condition				
Farms	111	25 (22.52)	1.184 (0.720–1.947)	0.506
Pet shops	112	22 (19.64)	0.956 (0.571–1.600)	0.863
Private breeding sites	380	75 (19.74)	0.921 (0.612–1.386)	0.693
Total	603	122 (20.23)		

^*^p < 0.05, ^**^p < 0.01.

To analyze the relationship between *Cryptosporidium* spp. infections and clinical manifestations in snakes, an additional 147 fecal samples from symptomatic snakes were collected. A general assessment of clinical symptoms was categorized into postprandial regurgitation, diarrhea gastrointestinal, and gastric bulge (Table 2).

**Table 2:** Associations between *Cryptosporidium* spp. and occurrence of clinical symptoms in snakes, as indicated by *χ*^2^ and Odds ratios analysis of data. A potential risk factor for infection was considered when *p* < 0.05 and OR > 1.

Clinical sign	*N*						Pathogens									
		*Cryptosporidium* spp.					*C. serpentis*					*C. varanii*				
		No. Positive (%)	*χ* ^2^	OR	95% CI	*p*	No. Positive (%)	*χ* ^2^	OR	95% CI	*p*	No. Positive (%)	*χ* ^2^	OR	95% CI	*p*
Regurgitation	59	48 (81.35)	5.122	2.618	1.195–5.737	0.016^*^	39 (66.10)	25.880	6.630	3.181–13.817	0.000^***^	9 (15.25)	8.989	0.273	0.119–0.624	0.003^*^
Diarrheic	56	44 (78.57)	2.498	1.989	0.921–4.294	0.080	16 (28.57)	4.288	0.447	0.219–0.909	0.038^*^	28 (50.00)	15.859	4.688	2.210–9.945	0.000^***^
Gastric bulge	32	11 (34.38)	24.847	0.131	0.055–0.310	0.000^***^	4 (12.50)	1.349	0.384	0.105–1.407	0.149	7 (21.88)	0.823	0.590	0.234–1.488	0.364
Total	147	103 (70.0)					59 (40.14)					44 (29.93)				

*N*: number of samples.

^*^p < 0.05, ^**^p < 0.01, ^***^p < 0.001.

Samples were stored in 2.5% potassium dichromate and transported to the laboratory, maintained at 4 °C until processing within one week.

### Microscopy screening

Fecal samples were mixed with phosphate-buffered saline (PBS) to make suspensions and purified through stainless steel screens with 63 μm porosity. We used Sheather’s discontinuous sucrose gradient centrifugation technique, as described by Arrowood, 2020. Staining of pretreated fecal samples was performed using dimethyl sulfoxide (DMSO)-modified acid-fast stain (Beijing Solarbio Science & Technology Co., Ltd., Beijing, China), as described by Arrowood and Sterling, 1987. Smears were subsequently examined using a bright-field microscope with 100 and 400 × magnification.

### DNA extraction and PCR analysis

All fecal samples were washed twice with centrifugation at 2000×*g* for 10 min with distilled water to remove potassium dichromate. Genomic DNA was extracted using a TIANamp Stool DNA Kit (Beijing TransGen Biotech Co., Ltd., Beijing, China) from approximately 200 mg of each fecal sample, according to the instructions of the manufacturer. Genomic DNA was eluted into 50 μL of elution buffer and stored at −40 °C until PCR amplification.

A nested PCR was conducted, targeting an approximately 830-base-pair (bp) fragment in the small-subunit (SSU) rRNA sequence to determine the *Cryptosporidium* species. The primary primers were F1 (5’-TTCTAGAGCTAATACATGCG-3′) and R1 (5′-CCCATTTCCTTCGAAACAGGA-3′), followed by F2 (5′-GGAAGGGTTGTATTATTAGATAAAG-3′) and R2 (5′-AAGGAGTAAGGAACAACCTCCA-3′) [46].

The amplification process was performed in 25 μL volumes, including 1 μL of template DNA or primary PCR product, 2.5 μL 10 × KOD-Plus PCR buffer, 2.5 μL dNTPs (2 nM), 1.5 μL MgSO_4_ (25 nM), 0.5 μL of each primer (25 nM), 16 μL double-distilled water, and 0.5 μL KOD-Plus amplification enzyme (1 unit/μL) (ToYoBo Co., Ltd., Osaka, Japan). There was an initial denaturation at 94 °C for 5 min, 35 cycles at 94 °C for 45 s, 55 °C for 45 s, 72 °C for 1 min, and a final extension at 72 °C for 7 min in PCR amplification. The secondary cycling conditions were identical to those used in the primary PCR. Both the positive control (DNA from the PCR-positive snake fecal samples) and negative control (1 μL double-distilled water replaced 1 μL template DNA) were used in each PCR amplification. The reactions were performed in an Applied Biosystems thermocycler. The final products from the amplification were analyzed through electrophoresis on 1% agarose gel by staining with ethidium bromide and observed under UV light.

### Cloning

A selection of 26 asymptomatic and 103 clinically symptomatic secondary PCR-positive products were cloned. PCR products were purified using a FastPure Gel DNA Extraction Mini Kit (Vazyme Biotech Co., Ltd., Nanjing, China), according to the manufacturer’s instructions. Positive products were cloned into 5 min^TM^ TA/Blunt-Zero Cloning Kit (Vazyme Biotech Co., Ltd.) and used to transform *Escherichia coli DH5α* cells. Clones were selected on LuriaBertani (LB) agar supplemented with 50 μg of ampicillin.mL^−1^. We successfully cloned 129 positive *E. coli DH5α*.

### Sequence and phylogenetic analysis

All positive clones underwent molecular analysis and genetic sequencing to identify the species of *Cryptosporidium.* These recombinant clones were sequenced unidirectionally at Tsingke Biotech Co., Ltd., Beijing, China. Nucleotide sequences were assembled with ChromasPro2.1.6 (https://www.technelysium.com.au/ChromasPro.html), and edited using BioEdit 7.1.3 (https://www.mbio.ncsu.edu/BioEdit/bioedit.html). Nucleotide sequences were aligned with the reference sequences from GenBank using ClustalX 2.1 software (https://www.clustal.org). Phylogenetic relationships among the species were evaluated using neighbor-joining analyses in MEGA 11 (https://www.megasoftware.net/), with branch reliability assessed by 1000 bootstrap replicates. A phylogenetic tree representing the *Cryptosporidium* spp. was then constructed. Nucleotide sequences generated in the study were submitted to GenBank under accession numbers PP088070–PP088103, PP101398–PP101411, and PP124552–PP124568 for the SSU rRNA gene.

### Statistical analysis

The Chi-square test, conducted using IBM SPSS Statistics 22 software (International Business Machines Corp., New York, NY, USA), was used to compare differences in *Cryptosporidium* spp. infection rates across different factors such as sex, age, species, living condition, and gastrointestinal clinical symptoms. Odds ratios (ORs) with 95% confidence intervals (CIs) were used to identify the risk factors associated with the occurrence of these pathogens in snakes. Statistical significance was established at *p* < 0.05.

## Results

### Prevalence and risk factors of *Cryptosporidium* spp. in random sampling

Among the 603 fecal samples collected, nested PCR identified 122 as positive for *Cryptosporidium* spp. (Table 1). The prevalence of *Cryptosporidium* spp. ranged from 6.25% to 40.00% in different provinces (Fig. 1). There was a slight difference in the infection *Cryptosporidium* spp. rates among PCR detection (20.23%, 122/603), Sheather’s sugar flotation detection (17.58%, 106/603, *χ*^2^ = 1.385, *p* = 0.239), and DMSO-modified acid-fast stain detection (18.24%, 110/603, *χ*^2^ = 0.769, *p* = 0.381).

The detection rate of *Cryptosporidium* spp. varied by age group (Table 1). The age group data analysis revealed that snakes aged ≥3 years (31.06%, 41/132) had a significantly higher detection rate than those 0–1 years (17.46%, 44/252; *χ*^2^ = 9.296, *p* = 0.002), and 1–2 years (13.67%, 19/139; *χ*^2^ = 8.419, *p* = 0.004). However, the difference in detection rates between snakes aged ≥3 years (31.06%, 41/132) and aged 2–3 years (22.5%, 18/80; *χ*^2^ = 1.817, *p* = 0.178) was not statistically significant. In contrast, there were no significant impacts on the infection rates of *Cryptosporidium* spp. in sex, species, and living conditions (*p* > 0.05) (Table 1). The difference in the infection rate of *Cryptosporidium* was not significant between males (21.17%, 58/274) and females (19.16%, 55/287, *χ*^2^ = 0.350, *p* = 0.554). In species groups, the Corn snake (23.36%, 25/107) showed a slightly significant difference in infection rate compared with the Pine snake (15.79%, 15/95, *χ*^2^ = 1.818, *p* = 0.178), Common garter snake (19.54%, 17/87, *χ*^2^ = 0.414, *p* = 0.520), California king snake (21.90%, 23/105, *χ*^2^ = 0.064, *p* = 0.800), Hognose snake (18.64%, 22/118, *χ*^2^ = 0.872, *p* = 0.350), and Black rat snake (21.98%, 20/91, *χ*^2^ = 0.054, *p* = 0.817). Furthermore, among the living condition groups that tested positive for *Cryptosporidium* spp., there was no significant difference in infection rates observed on farms (22.52%, 25/111), in pet shops (19.64%, 22/112, *χ*^2^ = 0.350, *p* = 0.554), and private breeding sites (19.74%, 75/380, *χ*^2^ = 0.350, *p* = 0.554).

Furthermore, odds ratios analysis revealed a risk factor involved in *Cryptosporidium* spp. transmission in snakes (Table 1). Snakes older than three years exhibited a significantly higher risk of *Cryptosporidium* spp. infection, with a prevalence of 31.06% (χ² = 12.278, OR = 2.196, *p* = 0.001), indicating particular vulnerability in this age group. Conversely, sex, species, and living conditions of snakes did not have significant impacts on infection rates of *Cryptosporidium* spp.

### Distribution of *Cryptosporidium* spp.

Successful species identification was achieved via sequence analysis in 129 samples. Two *Cryptosporidium* spp. were identified: *C. serpentis*, comprising 54.26% (70/129) of the samples, and *C. varanii*, accounting for 45.74% (59/129). Both *C. serpentis* and *C. varanii* were identified in snakes across a broad range of ages, sexes, and species. However, the *C. serpentis* W11 genotype was the only genotype identified in the Common garter snake.

**Figure 2:**
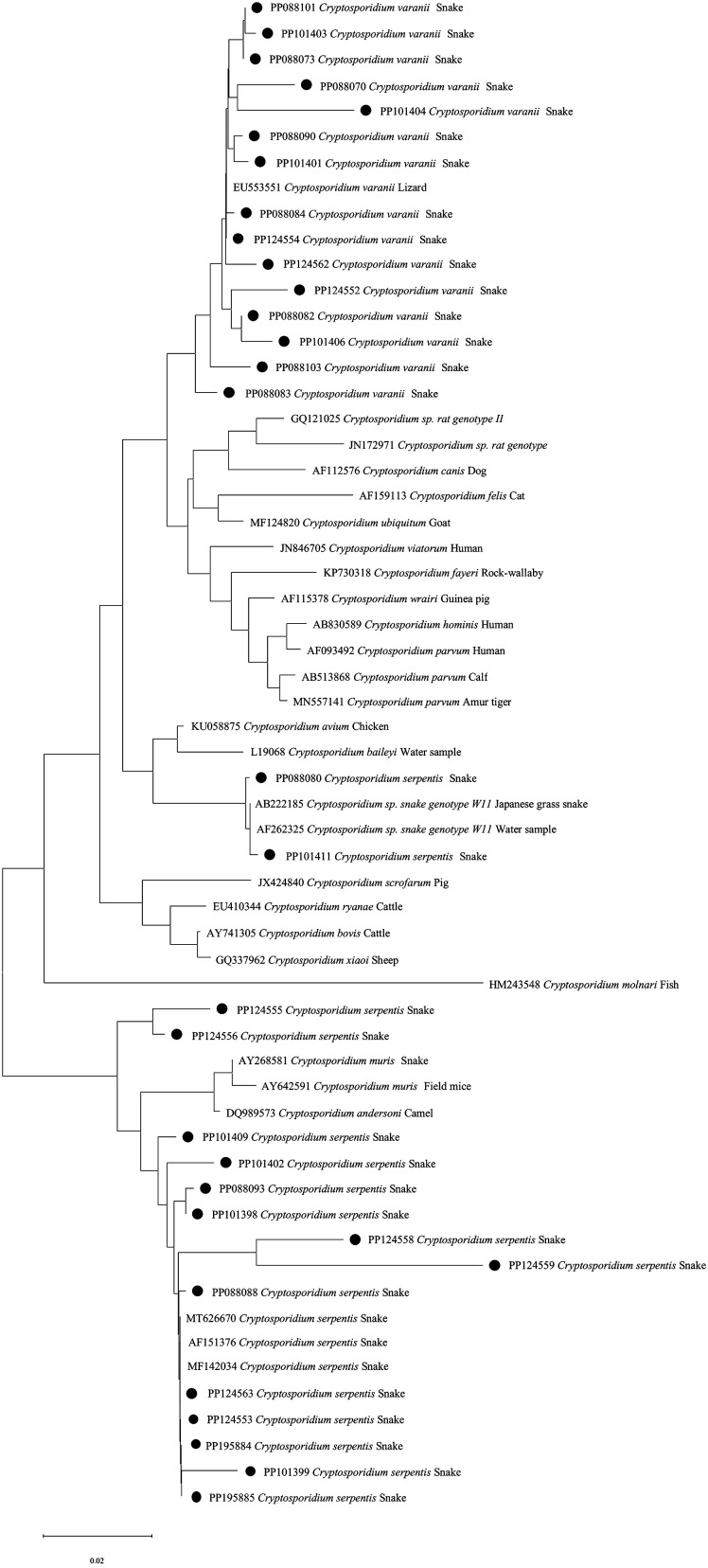
Phylogenetic relationships of *Cryptosporidium* spp. isolates in this study to other known *Cryptosporidium* spp. inferred by neighbor-joining analysis of the SSU rRNA gene. Sequences were retrieved from GenBank, aligned using ClustalW, and analyzed using MEGA 11 software. The neighbor-joining method was used to construct the trees from the Kimura-2-parameter model. Branch numbers represent percent bootstrapping values from 1000 replicates, with values of more than 50% shown in the tree. *Cryptosporidium* spp. found in this study are marked with a ●.

The SSU rRNA sequences of *C. serpentis* isolates identified were identical, exhibiting a T to C single nucleotide substitution and an additional A nucleotide compared with the reference sequence AF262325 [45] from stormwater samples in New York and AB222185 [28] from Japanese grass snake. For *C. varanii*, the SSU rRNA sequences matched the reference sequence EU553551 [45] from Black rat snake, including one single nucleotide substitution in one sample and 2–8 nucleotide deletions in 14 samples. Non-100% homology species were selected for phylogenetic analysis of SSU rRNA sequences, 15 of *C. serpentis* clustered together with AF151376 [26], and two species of *C. serpentis* were clustered in AF262325 and AB222185. In contrast, all *C. varanii* clustered together with EU553551 (Fig. 2).

### Association between *Cryptosporidium* spp. and clinical symptoms

Among the 147 fecal samples collected, 103 (70.0%) were positive for *Cryptosporidium* spp. (Table 2). Odds ratios analysis identified various risk factors involved in *Cryptosporidium* spp. transmission in snakes. Specifically, snakes that regurgitation (81.35%; *χ*^2^ = 5.122, OR = 2.618, *p* = 0.016) had a higher risk of *Cryptosporidium* spp. infection.

*Cryptosporidium serpentis* did not show higher prevalence (40.14%, 59/147) than *C. varanii* (29.93%, 44/147, *χ*^2^ = 3.362, *p* = 0.067). The chi-square analysis of clinical symptoms showed that infections of *C. varanii* and *C. serpentis* were significantly associated with diarrhea (OR = 4.688, 95% CI = 2.210–9.945, *χ*^2^ = 15.859, *p* < 0.001) and regurgitation (OR = 6.630, 95% CI = 3.181–13.817, *χ*^2^ = 25.880, *p* < 0.001), respectively. Additionally, *C. serpentis* infection is specifically associated with the occurrence of postprandial regurgitation (OR = 6.630, 95% CI = 3.181–13.817, *χ*^2^ = 25.880, *p* < 0.001) in symptomatic snakes (Table 2). In contrast, the occurrence of gastric bulge was not associated with *C. varanii* (OR = 0.590, 95% CI = 0.234–1.488, *χ*^2^ = 0.823, *p* = 0.364) and *C. serpentis* (OR = 0.384, 95% CI = 0.105–1.407, *χ*^2^ = 1.349, *p* = 0.149) (Table 2).

## Discussion

*Cryptosporidium* spp. infection is a significant cause of disease in wild and captive snakes [4, 47], potentially leading to fatality [18]. The present study investigated the prevalence of *Cryptosporidium* spp. in snake populations in China, with an average infection rate of 20.23% by nested PCR detection. While Sheather’s sugar flotation and DMSO-modified acid-fast stain detections have slightly lower sensitivity in the detection of *Cryptosporidium* spp. infection, they offer simpler and more effective veterinary clinical diagnosis [20]. Therefore, employing a multifaceted approach that integrates various diagnostic techniques remains essential for the comprehensive detection of *Cryptosporidium* spp. infection [32].

*Cryptosporidium* spp. has been reported from different countries worldwide, with molecular detection revealing infection rates ranging from 1.3 to 64.6% in snakes [3, 11, 14, 24, 28, 34, 38, 39, 46, 48, 50].

In this study, the overall *Cryptosporidium* spp. infection rate in snakes was 20.23%, which is lower than most rates reported in Japanese grass snakes in Japan (25.6%, 57/223) [28], and in Louisville Zoo and the National Zoological Park snakes in the United States (64.6%, 31/48) [47], but higher than that in Corn snakes in Austria (17.6%, 12/68) [38], and in 13 species of snakes in Wuhan, China (10.1%,15/149) [48]. The infection rate in captive snakes was 22.52%, which is higher than previously reported in south-western and northern China (1.97%, 12/609) [11], and Oriental rat snakes in Guangxi, China (3/141, 2.1%) [24], in 15 species of snakes in Kerala, India (10.2%,5/49) [1], and 7 species of snakes in Rio de Janeiro, Brazil (19.0%, 36/189) [29]. However, the infection rate in pet snakes (19.72%, 97/492) was lower than that in 8 snake species in Thailand (24.2%, 40/165) [50], 5 snake species in Spain (25.1%, 48/189) [34], and from 13 different genera of snakes in Italy (29.2%, 35/120) [14], a significantly lower prevalence of *Cryptosporidium* spp. than that in pet snakes from Italy (6/125, 4.8%) [39] and Beijing in China (17/273, 6.2%) [51]. Furthermore, sex, species, or living conditions were not risk factors for *Cryptosporidium* spp. in snakes, consistent with previous studies [51]. In contrast, advanced age emerged as a primary risk factor, particularly in snakes over three years of age. Therefore, the results of the present study suggest distinct transmission dynamics of *Cryptosporidium* spp. in snakes. Differences in *Cryptosporidium* spp. infection rates may be attributed to factors such as sampling times, feeding practices, and management models.

In this study including *Cryptosporidium* molecular characterization, results support the suggestion that *C. serpentis* and *C. varanii* are the most common *Cryptosporidium* species in snakes [14, 47]. We identified the infection of both *C. serpentis* and *C. varanii* in snakes, with *C. serpentis* being the dominant species in 70 of 129 *Cryptosporidium*-positive specimens. Interestingly, despite it having been reported previously in six species of snakes, this is the first report of *C. serpentis* genotype W11 in the Common garter snake and even in sympatrically distributed species that did not occur [28]. The positive Common garter snake found on farms indicates that *C. serpentis* genotype W11 does occur in China snake populations. In addition, *C. serpentis* has not only been reported in reptiles, but also in cows [12] and Alaskan caribou [43], highlighting its broad host range. The varied infection dynamics of *C. serpentis* among different hosts could be related to biological differences among *C. serpentis* strains. Consequently, more epidemiological data are needed to better understand the infection dynamics of *C. serpentis* across species of animals.

To date, limited molecular epidemiological studies of snake *Cryptosporidium* spp. have primarily focused on snakes without gastrointestinal clinical symptoms [38, 51]. This study is the first to specifically analyze the clinical symptoms of cryptosporidiosis and *Cryptosporidium* spp. via statistical analysis of epidemiological data on snakes in China. Results show that *C. serpentis* and *C. varanii* infection occurs in snakes during gastrointestinal clinical symptoms with a high infection rate, which identified *C. serpentis* and *C. varanii*, respectively, as the likely cause of regurgitation and diarrhea. In contrast, swelling of the stomach is not associated with *Cryptosporidium* spp. but rather with other pathogens, such as flagellates and enteropathogenic bacteria [44]. The clinical signs observed in some *Cryptosporidium-*positive snakes align with previously reported cases of chronic gastrointestinal disease [10, 25, 35], whereas snakes without clinical signs may remain in subclinical stages [31]. During the collection of samples from diseased snakes, several owners described cases of gastric swelling leading to sudden death. Although clinical symptoms vary depending on age, species, host immune status, and environmental conditions, our data analysis may reflect risk factors associated with clinical signs of cryptosporidiosis in snakes.

*Cryptosporidium* spp. infection seemingly correlates with hygiene standards and management practices [19], where poor management and unsanitary conditions result in a high risk for snakes. Manure serves as a critical reservoir for *Cryptosporidium* spp., with oocysts from infected animals leading to substantial environmental contamination [17, 36]. Oocyst resistance to many disinfectants and their survival in a wide temperature range (22–60 °C) facilitate persistent fecal-oral transmission [9, 27]. Furthermore, subclinically infected snakes may excrete oocysts, causing the transmission of the infection to their offspring if ingested by pregnant snakes through contaminated food and water [33]. Consequently, *Cryptosporidium* spp. infections can be reduced by paying keen attention to the snake’s external environment. Furthermore, drastic control measures including monitoring snakes regularly for *Cryptosporidium* spp., cutting off transmission by eliminating contact, quarantining, and euthanasia of infected snakes remain ways to avoid *Cryptosporidium* spp. infection [26, 47].

## Conclusion

In conclusion, the results of this study reveal high prevalence of *Cryptosporidium* spp. in asymptomatic snakes in China and old age as a risk factor for infections. Specifically, *C. serpentis* and *C. varanii* were identified as the predominant species in symptomatic snakes. Of note, these snake species are common in China and their close contact with humans poses a potential health risk to keepers. Mitigation of the effects of cryptosporidiosis requires the implementation of effective management strategies to minimize environmental contamination by infectious *Cryptosporidium* oocysts, while improving the health and welfare of snakes. Additionally, a large-scale survey of snake cryptosporidiosis is needed to better assess the public health significance of *Cryptosporidium* species.
